# Bibliometric analysis of ketogenic diet for pain based on dual databases: global trends and research hotspots from the Web of Science Core Collection and Scopus (2006–2025)

**DOI:** 10.3389/fnut.2026.1717096

**Published:** 2026-02-27

**Authors:** Mo Liao, Xinhui Cheng, Fei Liu, Xingjuan Xiong, Yongsheng Yu

**Affiliations:** Rehabilitation Center, Dazhou Integrated TCM & Western Medicine Hospital, Dazhou, Sichuan, China

**Keywords:** bibliometric analysis, dual-database approach, ketogenic diet, pain management, research hotspots

## Abstract

**Objective:**

The ketogenic diet (KD) has demonstrated beneficial effects on pain, yet no bibliometric study has systematically explored this association. This study aimed to evaluate global research trends and emerging hotspots on “ketogenic diet for pain” from 2006 to 2025, providing quantitative evidence and forward-looking perspectives for future basic and clinical research.

**Methods:**

Publications from 2006 to 2025 were retrieved from the Web of Science Core Collection (WoSCC) and Scopus databases. Bibliometric analyses were conducted using Bibliometrix (R package), VOSviewer, and CiteSpace to assess performance and generate visualizations.

**Results:**

This study included 328 eligible publications from the WoSCC and 786 from the Scopus database. From 2006 to 2021, the annual number of publications on KD–related pain research showed a sustained upward trend, followed by a phase of high-level fluctuation between 2022 and 2025. The United States emerged as the leading contributing country. An international collaboration network was formed, with the University of Basel (Switzerland) and Sapienza University of Rome (Italy) serving as central hubs. Nutrients published the largest number of articles and received widespread citations, whereas Cephalalgia was the most frequently cited journal. Nutrients and Epilepsia functioned as key academic collaboration hubs. Keyword co-occurrence and clustering analyses identified three major thematic domains: (1) the direct analgesic effects of the KD and its role in the management of chronic inflammatory pain; (2) the neuroprotective mechanisms of the KD and its synergistic analgesic effects when combined with pharmacological therapies; and (3) the improvement of comorbid mood disorders and the mitigation of metabolic risks associated with ketogenic dietary interventions. Current research hotspots focus on dietary strategies for migraine management, the application of KD in chronic pain conditions, and ketone body–mediated anti-inflammatory and neuromodulatory mechanisms.

**Conclusion:**

Research on the beneficial role of KD in pain management has attracted growing global attention and is expected to become an important direction in future pain management. This study provides a comprehensive bibliometric overview of the current status and research hotspots, offering valuable guidance for subsequent investigations.

## 1 Introduction

Pain, as recently defined by the International Association for the Study of Pain (IASP), is “an unpleasant sensory and emotional experience associated with, or resembling that associated with, actual or potential tissue damage” (1). It has become a major global health challenge. According to the Global Burden of Disease (GBD) 2019 analysis, pain-related conditions—particularly low back pain and headache—are the leading causes of years lived with disability (YLDs) worldwide (2). Globally, about one in five adults suffers from chronic pain, which not only impairs physical function and mental health but also imposes a substantial socioeconomic burden through high healthcare costs and reduced productivity (3).

Currently, pharmacological interventions remain the cornerstone of pain management, especially the use of nonsteroidal anti-inflammatory drugs (NSAIDs) and opioids. However, both classes of drugs present considerable risks with long-term use. A large individual participant data meta-analysis confirmed that traditional NSAIDs and COX-2 inhibitors significantly increase the risk of serious cardiovascular and gastrointestinal adverse events (4). Meanwhile, opioid misuse and addiction have escalated into a global public health crisis, creating a paradox between their strong analgesic effects and devastating addictive consequences, and thereby posing a critical challenge to global health (5). These limitations highlight the urgent need for safer and more effective non-pharmacological interventions.

Within this context, the ketogenic diet (KD), a distinctive metabolic therapy, has attracted growing interest for its potential in pain management. KD is characterized by very low carbohydrate intake, moderate protein consumption, and high fat intake, designed to mimic fasting and induce nutritional ketosis (6). Under this metabolic condition, the liver converts fatty acids into ketone bodies (primarily beta hydroxybutyrate (BHB) and acetoacetate), which can cross the blood–brain barrier and serve as an alternative energy source to glucose for the brain and other tissues (7). The therapeutic efficacy of KD was first established in refractory epilepsy, with a randomized controlled trial published in The Lancet Neurology demonstrating a significant reduction in seizure frequency among children (8).

In recent years, KD applications have expanded beyond epilepsy to a wide range of neurological and systemic conditions. Systematic reviews have highlighted its therapeutic potential in Alzheimer’s disease (9), its adjunctive role in oncology (10), and its benefits for metabolic syndrome (11). Regarding pain, a growing body of preclinical evidence suggests that KD may exert analgesic effects through multiple mechanisms. Proposed pathways include BHB acting as a signaling molecule to inhibit the NLRP3 inflammasome and attenuate inflammation (12); activation of PPAR-γ receptors to reduce neuroinflammation (13); modulation of the balance between the inhibitory neurotransmitter GABA and the excitatory neurotransmitter glutamate to suppress neuronal hyperexcitability (14); and enhancement of mitochondrial biogenesis alongside reduced reactive oxygen species (ROS) production to improve cellular energy metabolism and oxidative stress (7). Collectively, these findings provide a strong biological rationale for considering KD as a novel approach to pain management.

Bibliometrics, as a core methodology in scientometrics, enables systematic exploration of publication trends, emerging topics, interdisciplinary collaborations, and international research networks, thereby offering valuable insights into the evolution of scientific fields (15). In this study, we applied bibliometric methods to analyze literature on “ketogenic diet and pain” published between January 1, 2006, and December 31, 2025, in the Web of Science Core Collection (WoSCC) and Scopus. Using bibliometric and visualization techniques, we aimed to map research output, collaboration networks, and emerging hotspots. By synthesizing research progress systematically, this study not only advances disciplinary development but also provides an evidence base for clinical practice and health policy.

## 2 Materials and methods

### 2.1 Data collection

Data were retrieved from the WoSCC and Scopus on December 31, 2025. In the WoSCC, the following search strategy was applied: TS = (pain* OR ache* OR algia OR headache OR migraine*) AND TS = (“ketogenic diet*” OR “keto diet*” OR ketosis OR “Atkins diet*”). This search yielded 391 records. After restricting the publication period to January 1, 2006–December 31, 2025, 369 records remained. Limiting the document types to Article or Review reduced the number to 339 records, and further restriction to English-language publications resulted in 328 records. Duplicate records were identified and removed by matching titles or DOIs, and irrelevant studies were excluded through screening. Ultimately, 328 eligible publications were identified from WoSCC. All retrieved records were saved in plain text format and exported as full records, including cited references. In Scopus, the following search strategy was used: (TITLE-ABS-KEY(pain*) OR TITLE-ABS-KEY(ache*) OR TITLE-ABS-KEY(algia) OR TITLE-ABS-KEY(headache) OR TITLE-ABS-KEY(migraine*)) AND (TITLE-ABS-KEY(“keto diet*”) OR TITLE-ABS-KEY(“ketogenic diet*”) OR TITLE-ABS-KEY(ketosis)). This search initially identified 992 records. After limiting the publication years to PUBYEAR > 2005 and PUBYEAR < 2026, 910 records remained. Restriction to Article or Review document types reduced the number to 825 records, and further limitation to English-language publications yielded 786 records. Duplicates were removed by matching titles or DOIs, and irrelevant records were excluded through screening, resulting in a final set of 786 eligible publications. All Scopus records were saved in CSV format and exported as full records, including cited references.

### 2.2 Data analysis

In this study, we adopted a previously established methodological framework (16). Annual publication trends were analyzed using Origin 2018. In addition, R software (version 4.5.1) was employed in combination with the bibliometrix package (version 4.0^1^) (17), VOSviewer (version 1.6.20) (18), and CiteSpace (version 6.1.4) (19). The bibliometrix package was used for the visual analysis and mapping of scientific knowledge, while VOSviewer was applied to visualize co-authorship networks at the country and institutional levels, co-citation relationships among source documents, and keyword co-occurrence patterns.

In the WoSCC and Scopus datasets, the minimum publication threshold for countries and institutions in the co-authorship network analysis was set at ≥5 to balance network completeness and interpretability, excluding entities with sporadic outputs while retaining those with sustained research activity. Sensitivity analyses using alternative thresholds of ≥3 and ≥10 publications showed that the overall network topology, core collaboration clusters, and leading countries and institutions remained highly consistent, with only minor variations among peripheral nodes, indicating that the main collaboration patterns were robust to threshold changes. In the co-citation analysis, the minimum citation frequency was set at ≥20, and sensitivity tests using ≥15 and ≥30 citations demonstrated stable core reference clusters and knowledge structures, with only limited changes in low-citation references, supporting the robustness of this threshold. For keyword co-occurrence analysis, a minimum occurrence threshold of ≥5 was applied to capture stable and representative research themes, and additional analyses using ≥3 and ≥7 occurrences yielded largely consistent research hotspots and thematic structures, with differences mainly confined to low-frequency peripheral keywords. Keywords such as “ketogenic diet,” “pain,” and their synonyms were excluded from the analysis. Journal impact factors (IFs) were retrieved from the 2024 Journal Citation Reports (JCR).

## 3 Results

### 3.1 Overview of research on the KD for pain

**Figure 1:**
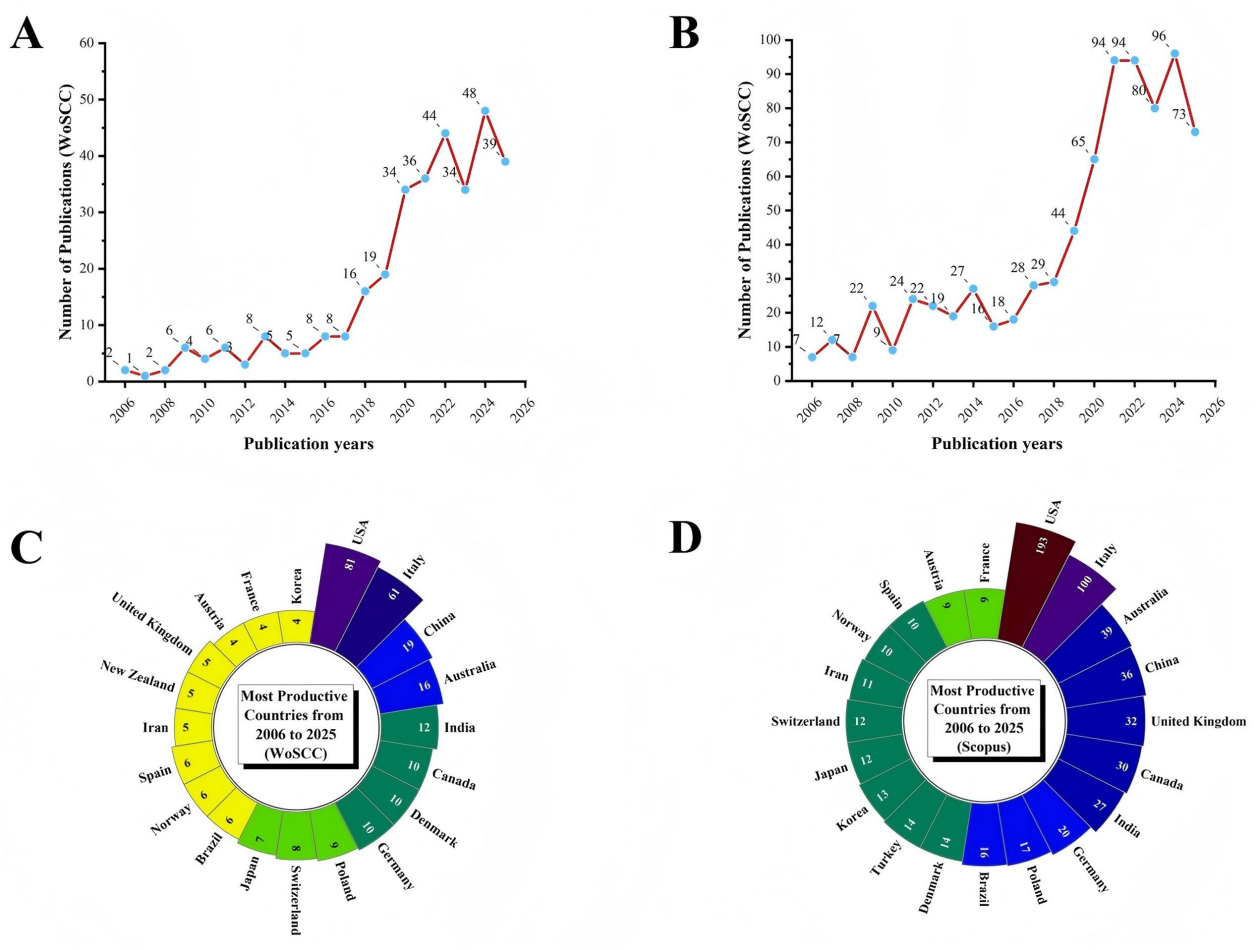
Annual publication trends on the relationship between KD and pain from 2006 to 2025. **(A)** Annual publication trends in WoSCC. **(B)** Annual publication trends in Scopus. **(C)** Distribution of corresponding authors’ countries in WoSCC. **(D)** Distribution of corresponding authors’ countries in Scopus.

A total of 328 unique records were retrieved from the WoSCC database. From 2006 to 2021, the number of publications related to the KD and pain showed a sustained upward trend. In contrast, during the period from 2022 to 2025, annual publication output remained at a relatively high level but exhibited moderate year-to-year fluctuations, without a clear unidirectional increasing or decreasing trend (Figure 1A). In the Scopus database, 786 unique records were identified. Between 2006 and 2021, publication output in this field increased steadily, indicating a continuous rise in research attention. Since 2022, annual publications have entered a relatively stable phase characterized by mild fluctuations following the earlier growth trend (Figure 1B). The consistent patterns observed across both databases suggest that research on the KD and pain experienced rapid development in its early stage and has gradually transitioned into a more stable phase in recent years.

**Figure 2:**
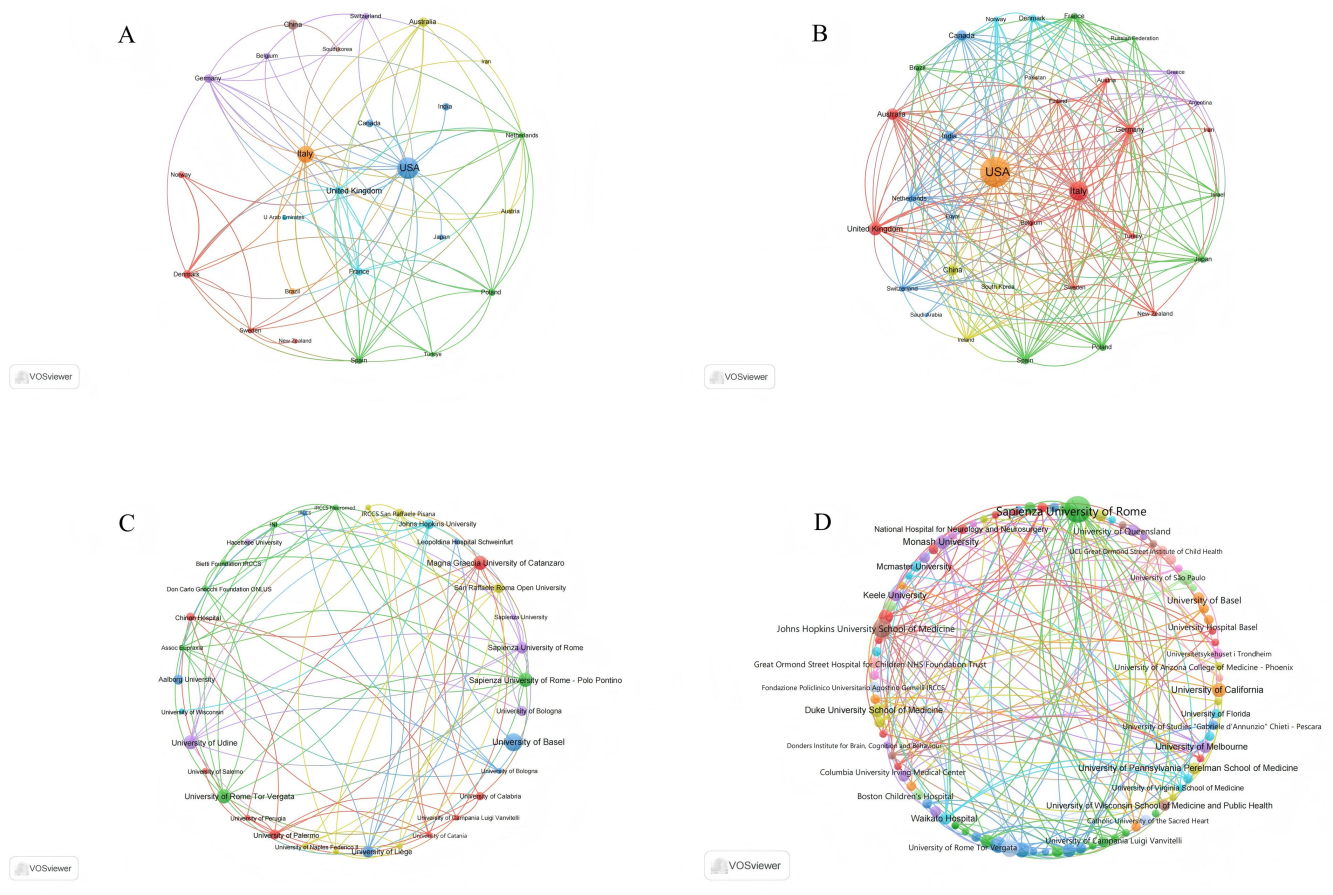
Global mapping of countries regions and institutions in KD for pain research from 2006 to 2025. **(A)** Geospatial collaboration network of countries based on WoSCC. **(B)** Geospatial collaboration network of countries based on Scopus. **(C)** Inter-institutional collaboration network derived from WoSCC. **(D)** Inter-institutional collaboration network derived from Scopus.

Analysis of contributing countries showed that in the WoSCC database, the United States (United States; *n* = 81) was the leading publishing country, followed by Italy (*n* = 61), China (*n* = 19), Australia (*n* = 16), and India (*n* = 12). In addition, 17 publications from the United States involved international collaboration, accounting for 21% of its total output, while 20 publications from Italy involved multinational collaboration, representing 32.8%. These findings indicate that the United States and Italy serve as major hubs of international collaboration in this field (Table 1; Figures 1C, 2A). Similarly, in the Scopus database, the United States (*n* = 193) ranked first in publication output, followed by Italy (*n* = 100), Australia (*n* = 39), China (*n* = 36), and the United Kingdom (*n* = 32). Among United States publications, 29 involved international collaboration, accounting for 15% of the total, further confirming the United States as a central collaborative hub in this research area (Table 1; Figures 1D, 2B). Overall, the United States not only leads in publication volume but also demonstrates broader and more extensive international collaboration networks. The leading position of the United States in research on KD–based pain management can be attributed to its strong comprehensive research capacity. First, advanced basic research in neuroscience provides a solid theoretical foundation for elucidating the analgesic mechanisms of the KD, such as modulation of neurotransmitter systems and attenuation of neuroinflammation. Second, the urgent opioid crisis has prompted national policy support and funding shifts toward non-pharmacological therapies, thereby facilitating large-scale clinical trials. Moreover, well-established clinical translation frameworks and a strong tradition of interdisciplinary collaboration have further accelerated the progression from basic discoveries to clinical application.

**Table 1:** Most relevant countries by corresponding authors.

Country	Articles	MCP	MCP %
WoSCC			
United States	81	17	21
Italy	61	20	32.8
China	19	2	10.5
Australia	16	5	31.3
India	12	1	8.3
Canada	10	2	20
Denmark	10	8	80
Germany	10	2	20
Poland	9	0	0
Switzerland	8	3	37.5
Japan	7	0	0
Brazil	6	1	16.7
Norway	6	5	83.3
Spain	6	2	33.3
Iran	5	2	40
New Zealand	5	2	40
United Kingdom	5	2	40
Austria	4	0	0
France	4	2	50
South Korea	4	1	25
Scopus			
United States	193	29	15
Italy	100	22	22
Australia	39	11	28.2
China	36	3	8.3
United Kingdom	32	9	28.1
Canada	30	5	16.7
India	27	2	7.4
Germany	20	8	40
Poland	17	2	11.8
Brazil	16	4	25
Denmark	14	7	50
Turkey	14	1	7.1
Korea	13	0	0
Japan	12	0	0
Switzerland	12	5	41.7
Iran	11	2	18.2
Norway	10	7	70
Spain	10	2	20
Austria	9	2	22.2
France	9	3	33.3

Institutional analysis further revealed that in the WoSCC database, the University of Basel (Switzerland; *n* = 9) was the most productive institution. In the Scopus database, Sapienza University of Rome (Italy; *n* = 193) ranked first in publication output. With respect to collaboration networks, Sapienza University of Rome - Polo Pontino served as a major collaborative hub in the WoSCC database (Degree = 13; Weighted Degree = 20), whereas Sapienza University of Rome occupied a similar central position in the Scopus database (Degree = 16; Weighted Degree = 32) (Table 2; Figures 2C,D). Collectively, the University of Basel and Sapienza University of Rome not only demonstrate high publication productivity but also maintain extensive international collaboration networks in this field.

**Table 2:** Most relevant affiliations of the relationship on KD for pain.

Affiliation	Articles (*n*)	Degree	Weighted degree
WoSCC			
University of Basel	9	2	4
Magna Graecia University of Catanzaro	7	9	18
Sapienza University of Rome - Polo Pontino	7	13	20
University of Rome Tor Vergata	7	10	21
University of Udine	7	8	15
Sapienza University of Rome	6	11	13
University of Liège	6	8	10
Aalborg University	5	1	1
Johns Hopkins University	5	9	10
San Raffaele Roma Open University	5	7	13
Udine University Hospital	5	3	8
University of Palermo	5	6	15
Centre Hospitalier de Chinon	4	5	14
IRCCS San Raffaele Pisana	4	9	11
University of Calabria	4	5	6
Associazione Eupraxia	3	10	14
Fondazione Don Carlo Gnocchi ONLUS	3	5	11
Fondazione IRCCS Istituto Neurologico Carlo Besta	3	5	11
Hacettepe University	3	1	1
University of Basel	3	7	12
Scopus			
Sapienza University of Rome	12	16	32
Johns Hopkins University School of Medicine	10	16	19
University of Rome Tor Vergata	9	9	16
University of Salerno	9	4	8
University of São Paulo	9	3	4
University of Pavia	9	7	10
University of Copenhagen	8	6	7
Monash University	8	9	15
University of California	7	2	4
Duke University School of Medicine	6	7	8
University of Milan	6	8	8
University of Udine	6	6	15
Keele University	6	8	14
Children‘s Hospital of Philadelphia	6	1	2
University of Queensland	6	3	3
University of Melbourne	6	14	19
University of Pennsylvania Perelman School of Medicine	5	3	5
Waikato Hospital	5	7	10
Aalborg University	5	2	3
Boston Children’s Hospital	5	1	1

### 3.2 Journal analysis and visualization

To evaluate journal influence, Bibliometrix was used for performance analysis, with ggplot2 for visualization and VOSviewer for co-citation mapping.

**Figure 3:**
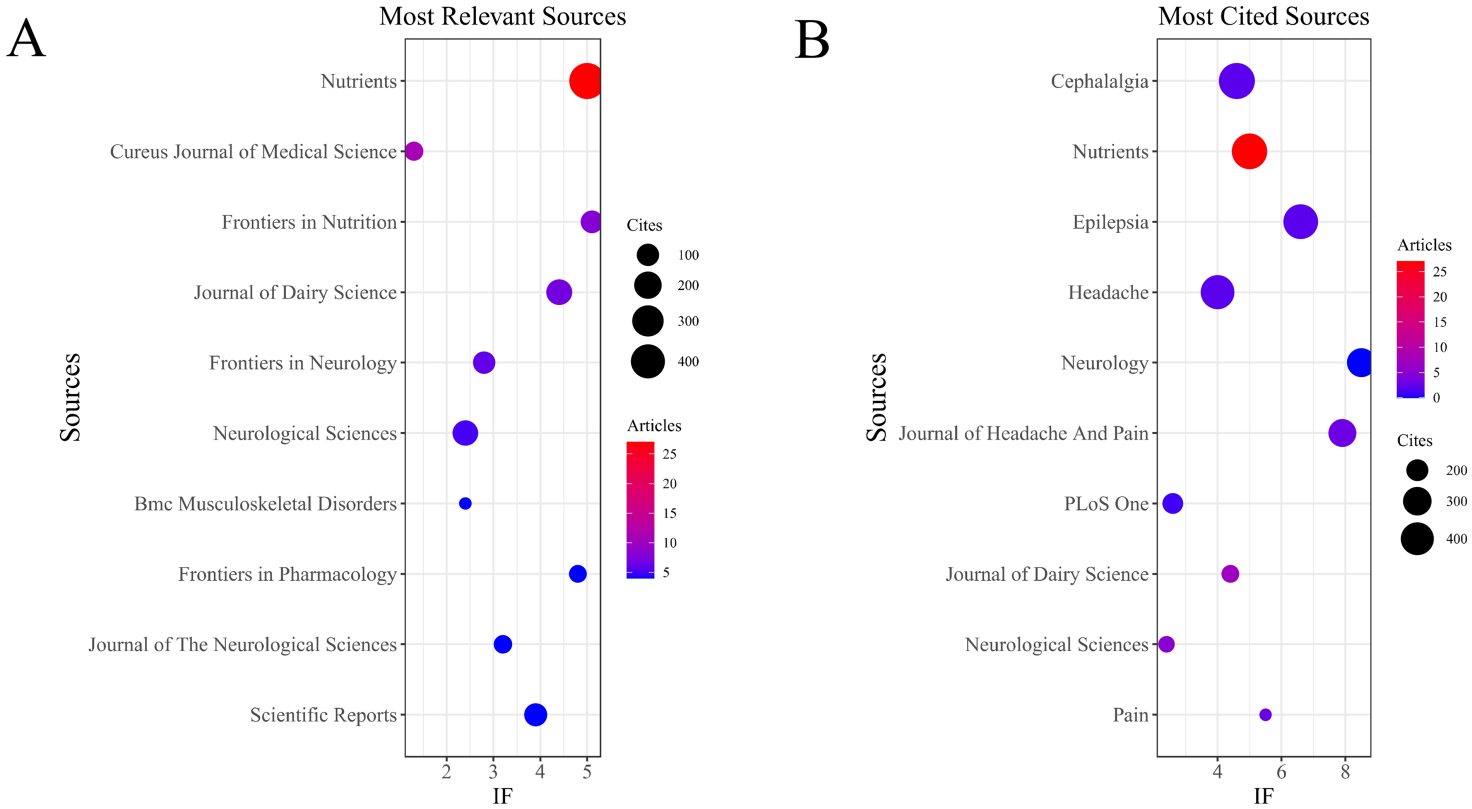
Journal with the largest number of articles published and the journal with the largest number of citations (WoSCC). **(A)** Journal with the largest number of articles published. **(B)** Journals with the largest number of citations.

In WoSCC, a total of 328 publications were retrieved, distributed across 208 academic journals (see Supplementary material S1). As shown in Table 3 and Figure 3A, Nutrients (*n* = 27; IF = 5.0) was the most productive journal, followed by Cureus Journal of Medical Science (*n* = 11; IF = 1.3), Frontiers in Nutrition (*n* = 8; IF = 5.1), Journal of Dairy Science (*n* = 7; IF = 4.4), and Frontiers in Neurology (*n* = 6; IF = 2.8). Table 4 and Figure 3B present the most frequently cited journals, including Cephalalgia (*n* = 486; IF = 4.6), Nutrients (*n* = 466; IF = 5.0), Epilepsia (*n* = 447; IF = 6.6), Headache (*n* = 423; IF = 4.0), and Neurology (*n* = 306; IF = 8.5).

**Table 3:** Top 10 journals with the most published articles.

WoSCC				Scopus		
Sources	Documents	Cites	IF (2004)	Sources	Documents	IF (2004)
Nutrients	27	466	5	Nutrients	48	5
Cureus Journal of Medical Science	11	56	1.3	Bmj Case Reports	14	0.5
Frontiers in Nutrition	8	103	5.1	Epilepsia	14	6.6
Journal of Dairy Science	7	164	4.4	Current Treatment Options in Neurology	13	1.8
Frontiers in Neurology	6	101	2.8	Epilepsy and Behavior	12	2.3
Neurological Sciences	5	156	2.4	Frontiers in Neurology	9	2.8
Bmc Musculoskeletal Disorders	4	21	2.4	Pediatric Neurology	9	2.1
Frontiers in Pharmacology	4	42	4.8	European Journal of Paediatric Neurology	8	2.3
Journal of the Neurological Sciences	4	50	3.2	Nutritional Neuroscience	8	3.6
Scientific Reports	4	109	3.9	Seizure	8	2.8

**Table 4:** Top 10 journals with the most cited journals (WoSCC).

Sources	Cites	Documents	IF (2024)
Cephalalgia	486	2	4.6
Nutrients	466	27	5
Epilepsia	447	2	6.6
Headache	423	2	4
Neurology	306	0	8.5
Journal of Headache and Pain	290	3	7.9
PLoS One	188	1	2.6
Journal of Dairy Science	164	7	4.4
Neurological Sciences	156	5	2.4
Pain	146	3	5.5

In Scopus, 786 publications were identified, spanning 426 academic journals (see Supplementary material S2). As summarized in Table 3, Nutrients (*n* = 48; IF = 5.0) remained the leading publishing journal, followed by BMJ Case Reports (*n* = 14; IF = 0.5), Epilepsia (*n* = 14; IF = 6.6), Current Treatment Options in Neurology (*n* = 13; IF = 1.8), and Epilepsy and Behavior (*n* = 12; IF = 2.3).

**Figure 4:**
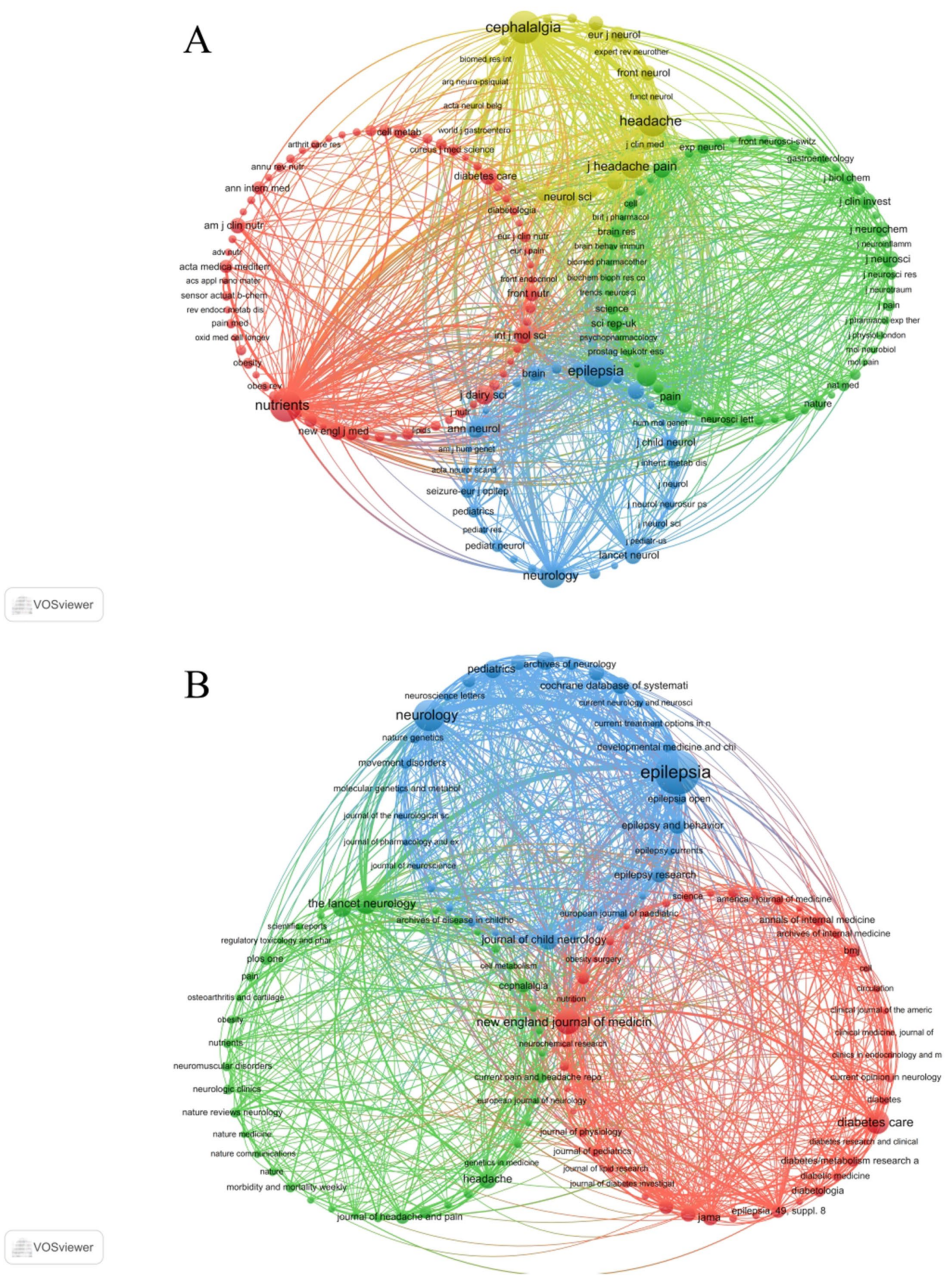
Co-cited journals related to KD for pain. **(A)** Journal co-citation network based on WoSCC. **(B)** Journal co-citation network based on Scopus.

Notably, the co-cited journal networks derived from the WoSCC and Scopus databases (Figures 4A,B) consistently identified Nutrients and Epilepsia as central hubs within the journal co-citation structure.

Overall, these findings collectively underscore the pivotal roles of Nutrients, Cephalalgia, and Epilepsia in advancing research on the KD and pain.

### 3.3 Most cited references and citation bursts

In WoSCC, the 20 most cited papers each received over 90 citations, spanning 19 journals (Table 5), indicating a relatively dispersed citation landscape. Highly influential works included “The ketogenic diet as a treatment paradigm for diverse neurological disorders,” “β-Hydroxybutyrate deactivates neutrophil NLRP3 inflammasome to relieve gout flares,” and “A ketogenic diet suppresses seizures in mice through adenosine A₁receptors.”

**Table 5:** Top 20 cited references related to the relationship on KD for pain (WoSCC).

Paper	Title	Total citations
STAFSTROM CE, 2012, FRONT PHARMACOL	The ketogenic diet as a treatment paradigm for diverse neurological disorders	327
GOLDBERG EL, 2017, CELL REP	β-Hydroxybutyrate Deactivates Neutrophil NLRP3 Inflammasome to Relieve Gout Flares	309
MASINO SA, 2011, J CLIN INVEST	A ketogenic diet suppresses seizures in mice through adenosine A₁ receptors	222
PHILLIPS MCL, 2018, MOVEMENT DISORD	Low-fat versus ketogenic diet in Parkinson’s disease: A pilot randomized controlled trial	204
ZUCCOLI G, 2010, NUTR METAB	Metabolic management of glioblastoma multiforme using standard therapy together with a restricted ketogenic diet: Case Report	191
BOISON D, 2017, CURR OPIN NEUROL	New insights into the mechanisms of the ketogenic diet	185
DE GIORGIS V, 2013, SEIZURE-EUR J EPILEP	GLUT1 deficiency syndrome 2013: current state of the art	144
NIJS J, 2014, EXPERT OPIN PHARMACO	Treatment of central sensitization in patients with ‘unexplained’ chronic pain: an update	144
RUSKIN DN, 2009, PLOS ONE	Reduced pain and inflammation in juvenile and adult rats fed a ketogenic diet	134
HINDIYEH NA, 2020, HEADACHE	The Role of Diet and Nutrition in Migraine Triggers and Treatment: A Systematic Literature Review	130
KOH S, 2020, EPILEPSY RES	Ketogenic diet and Neuroinflammation	122
DYNKA D, 2022, NUTRIENTS	The Role of Ketogenic Diet in the Treatment of Neurological Diseases	120
GAZERANI P, 2020, NUTRIENTS	Migraine and Diet	116
JAHROMI SR, 2019, J HEADACHE PAIN	Association of diet and headache	115
MASINO SA, 2009, CURR NEUROPHARMACOL	Adenosine, ketogenic diet and epilepsy: the emerging therapeutic relationship between metabolism and brain activity	105
LANS C, 2007, J ETHNOBIOL ETHNOMED	Ethnoveterinary medicines used for ruminants in British Columbia, Canada	101
DI LORENZO C, 2015, EUR J NEUROL	Migraine improvement during short lasting ketogenesis: a proof-of-concept study	100
FOND G, 2013, PSYCHIAT RES	Fasting in mood disorders: neurobiology and effectiveness. A review of the literature	98
MCDONALD TJW, 2018, NEUROTHERAPEUTICS	Ketogenic Diets for Adult Neurological Disorders	95
MICHALSEN A, 2013, FORSCH KOMPLEMENTMED	Fasting therapy for treating and preventing disease - current state of evidence	92

In Scopus, the top 20 articles each received more than 190 citations across 17 journals (Table 6). Seminal works included “Aging and aging-related diseases: from molecular mechanisms to interventions and treatments,” “The treatment of super-refractory status epilepticus: a critical review of available therapies and a clinical treatment protocol,” and “The Structure and Function of the Na, K-ATPase Isoforms in Health and Disease.”

**Table 6:** Top 20 cited references related to the relationship on KD for pain (Scopus).

Paper	Title	Total citations
GUO J, 2022, SIGNAL TRANSDUCT TARGET THER	Aging and aging-related diseases: from molecular mechanisms to interventions and treatments	1,080
SHORVON S, 2011, BRAIN	The treatment of super-refractory status epilepticus: a critical review of available therapies and a clinical treatment protocol	567
CLAUSEN MV, 2017, FRONT PHYSIOL	The Structure and Function of the Na, K-ATPase Isoforms in Health and Disease	415
STAFSTROM CE, 2012, FRONT PHARMACOL	The ketogenic diet as a treatment paradigm for diverse neurological disorders	375
WESTMAN EC, 2008, NUTR METAB	The effect of a low-carbohydrate, ketogenic diet versus a low-glycemic index diet on glycemic control in type 2 diabetes mellitus	364
BURNSTOCK G, 2011, PROG NEUROBIOL	Purinergic signalling: from normal behavior to pathological brain function	358
GOLDBERG EL, 2017, CELL REP	β-Hydroxybutyrate Deactivates Neutrophil NLRP3 Inflammasome to Relieve Gout Flares	322
PARIKH S, 2009, CURR TREAT OPTIONS NEUROL	PARIKH S, 2009, CURR TREAT OPTIONS NEUROL	294
ZHU H, 2022, SIGNAL TRANSDUCT TARGET THER	Ketogenic diet for human diseases: the underlying mechanisms and potential for clinical implementations	282
CLARKE K, 2012, REGUL TOXICOL PHARMACOL	Kinetics, safety and tolerability of (R)-3-hydroxybutyl (R)-3-hydroxybutyrate in healthy adult subjects	279
JOE E, 2019, BMJ	Cognitive symptoms of Alzheimer’s disease: clinical management and prevention	273
MASINO SA, 2011, J CLIN INVEST	A ketogenic diet suppresses seizures in mice through adenosine A₁ receptors	244
DMITRIEVA-POSOCCO O, 2022, NATURE	β-Hydroxybutyrate suppresses colorectal cancer	235
PHILLIPS MCL, 2018, MOV DISORD	Low-fat versus ketogenic diet in Parkinson’s disease: A pilot randomized controlled trial	226
ZUCCOLI G, 2010, NUTR METAB	Metabolic management of glioblastoma multiforme using standard therapy together with a restricted ketogenic diet: Case Report	217
GANO LB, 2014, J LIPID RES	Ketogenic diets, mitochondria, and neurological diseases	213
KLEPPER J, 2020, EPILEPSIA OPEN	Glut1 Deficiency Syndrome (Glut1DS): State of the art in 2020 and recommendations of the international Glut1DS study group	209
BOISON D, 2017, CURR OPIN NEUROL	New insights into the mechanisms of the ketogenic diet	209
GROSS EC, 2019, NAT REV NEUROL	The metabolic face of migraine - from pathophysiology to treatment	198
BARAÑANO KW, 2008, CURR TREAT OPTIONS NEUROL	The ketogenic diet: uses in epilepsy and other neurologic illnesses	196

**Figure 5:**
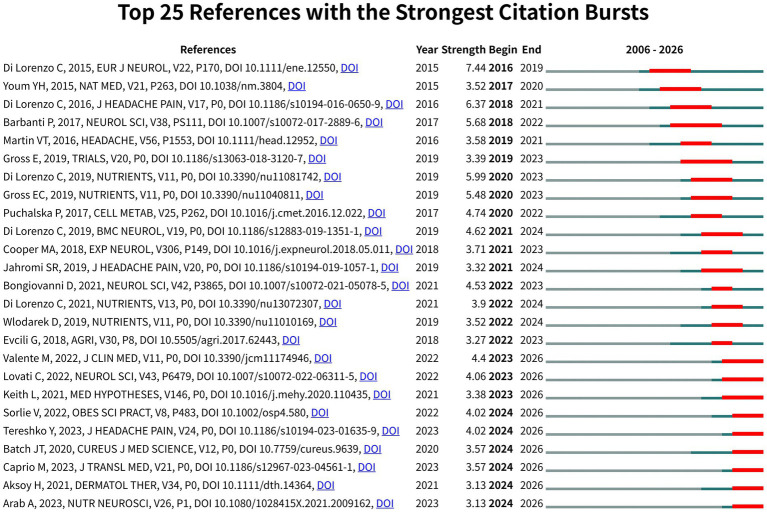
Top 25 references with the strongest citation bursts in KD for pain (WoSCC).

CiteSpace analysis revealed 25 citation bursts in WoSCC (Figure 5; Supplementary material S3). The strongest bursts were observed for: (1) Diet transiently improves migraine in two twin sisters: possible role of ketogenesis? (Strength = 7.44), (2) Cortical functional correlates of responsiveness to short-lasting preventive intervention with KD in migraine: a multimodal evoked potentials study (Strength = 6.37), (3) A randomized double-blind, cross-over trial of very low-calorie diet in overweight migraine patients: a possible role for ketones? (Strength = 5.99). Together, the most cited works and citation bursts highlight three main research directions: (1) Associations between adherence to Mediterranean dietary pattern and frequency, duration, and severity of migraine headache: A cross-sectional study; (2) Cause and management of lipedema-associated pain; (3) Very-low-calorie ketogenic diet vs. hypocaloric balanced diet in the prevention of high-frequency episodic migraine: the EMIKETO randomized, controlled trial.

Based on the integrated analysis of cited references and citation bursts, current research in this field is primarily focused on metabolic intervention strategies centered on the KD and ketone bodies, systematically investigating their therapeutic potential, underlying mechanisms, and pathways to clinical translation across a wide range of conditions, from neurological disorders (e.g., epilepsy and migraine) to inflammatory diseases (e.g., gout).

### 3.4 Keyword clusters and thematic evolution

**Figure 6:**
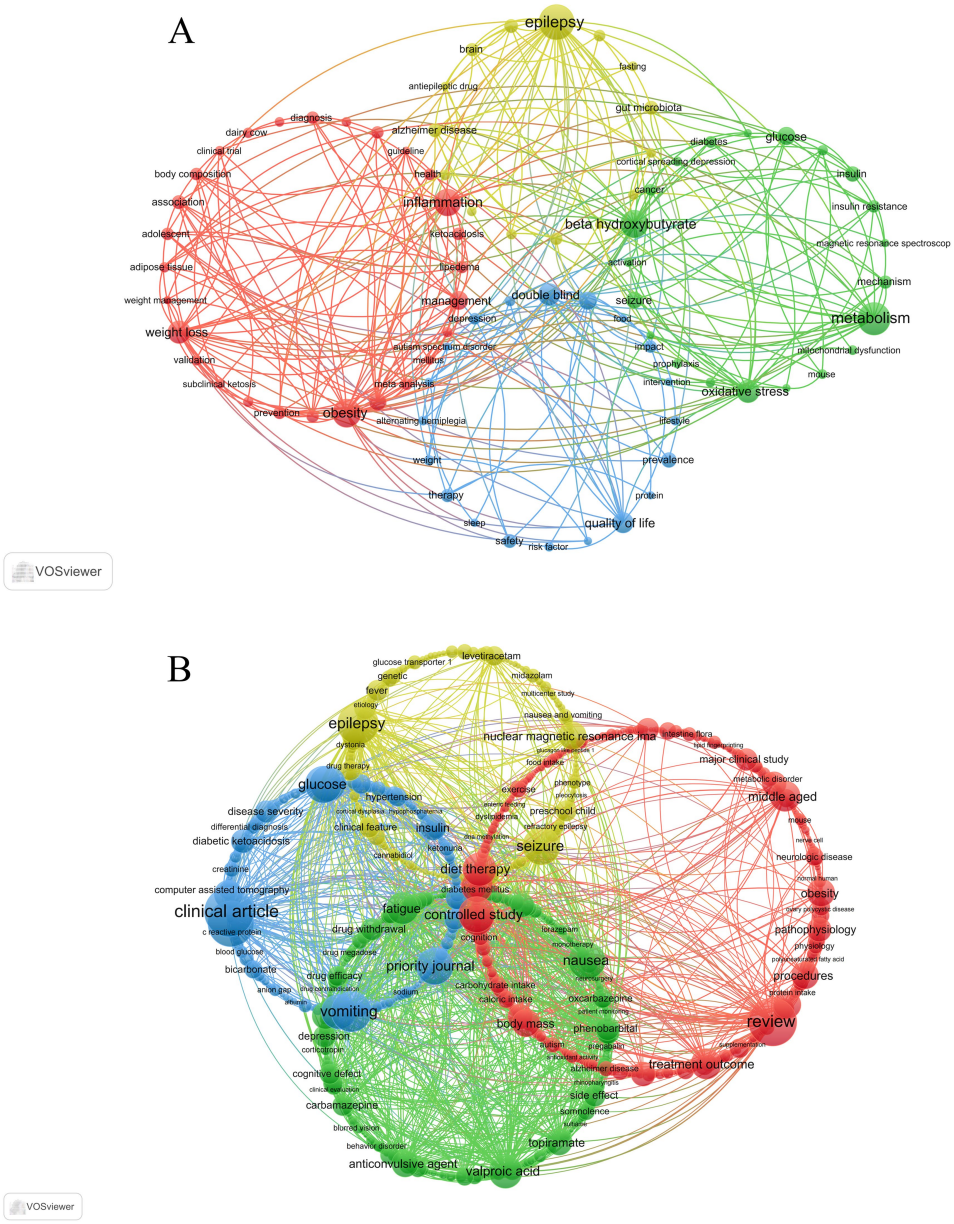
Keyword co-occurrence map of publications in KD for pain. **(A)** Keyword co-occurrence analysis based on WoSCC. **(B)** Keyword co-occurrence analysis based on Scopus.

Keyword clustering is essential for rapidly identifying major research themes and directions within a field. Prior to conducting the keyword clustering analysis, the proportion of epilepsy-related publications within the overall dataset was quantitatively assessed. In the WoSCC dataset, 91 publications (27.7%) were related to epilepsy, whereas in the Scopus dataset, 303 publications (38.6%) focused on epilepsy. These results indicate that, although epilepsy represents an important application area of the ketogenic diet, it does not dominate the literature, providing essential context for the subsequent keyword clustering and research hotspot analyses. In this study, VOSviewer identified 84 keywords from the WoSCC database. The top 20 keywords with more than 10 occurrences are listed in Table 7, highlighting the main research focuses. Epilepsy (*n* = 43) was the most frequent keyword, followed by Metabolism (*n* = 38), Obesity (*n* = 31), Beta hydroxybutyrate (*n* = 30), and Inflammation (*n* = 29). Cluster analysis revealed four distinct clusters (Figure 6A). (1) The red cluster (28 keywords) represents studies on the alleviation of chronic inflammatory and osteoarthritic pain through anti-inflammatory effects, metabolic regulation, and weight control induced by the KD. Core keywords included “inflammation,” “osteoarthritis,” “obesity,” and “body composition.” (2) The green cluster (21 keywords) focuses on the mechanisms of KD intervention in neuropathic pain, emphasizing the role of ketone body metabolism in reducing oxidative stress and improving energy metabolism to modulate neural function and pain. Representative keywords included “neuropathy,” “oxidative stress,” “metabolism,” and “beta hydroxybutyrate.” (3) The blue cluster (20 keywords) highlights the non-pharmacological value of the ketogenic diet in the comprehensive management of chronic pain accompanied by emotional disorders and reduced quality of life. Common keywords included “depression,” “quality of life,” “therapy,” “intervention,” and “lifestyle.” (4) The yellow cluster (15 keywords) reflects research on the therapeutic potential of the KD in episodic and central pain conditions, such as migraine and alternating hemiplegia, with keywords including “migraine,” “alternating hemiplegia,” “brain,” and “seizure.” All identified keywords are provided in Supplementary material S4. Overall, these clusters indicate that the role of the KD in pain research extends beyond direct analgesia and involves metabolic regulation, neuroprotection, and improvements in emotional status and quality of life.

**Table 7:** Top 20 keywords related to the relationship on KD for pain.

Words	Occurrences
WoSCC	
Epilepsy	43
Metabolism	38
Obesity	31
Beta Hydroxybutyrate	30
Inflammation	29
Oxidative Stress	23
Weight Loss	23
Double Blind	22
Quality of Life	20
Management	17
Efficacy	16
Glucose	16
Nutrition	15
Seizure	14
Insulin	12
Prevalence	12
Alzheimer Disease	11
Association	11
Caloric Restriction	11
Gut Microbiota	11
Scopus	
Clinical Article	252
Review	239
Case Report	225
Epilepsy	193
Vomiting	190
Seizure	170
Priority Journal	166
Glucose	165
Nausea	163
Controlled Study	162
Glucose Blood Level	154
Abdominal Pain	153
Valproic Acid	143
Diet Therapy	141
Fatigue	134
Diarrhea	131
Middle Aged	127
Treatment Outcome	126
Constipation	124
Complication	114

Using VOSviewer, 976 keywords were identified from the Scopus database. The top 20 keywords with more than 113 occurrences are presented in Table 7, reflecting the main research emphases. Clinical Article (*n* = 252) was the most frequent keyword, followed by Review (*n* = 239), Case Report (*n* = 225), Epilepsy (*n* = 193), and Vomiting (*n* = 190). Four clusters were identified (Figure 6B). (1) The red cluster (383 keywords) mainly focuses on the direct analgesic effects of very low-calorie KD in pain syndromes such as migraine and arthritis. Representative keywords included “very low calorie ketogenic diet,” “diet, carbohydrate-restricted,” “pain intensity,” “arthralgia,” “migraine disorders,” and “migraine with aura.” (2) The yellow cluster (259 keywords) emphasizes the synergistic effects of KD and antiepileptic drugs in the management of neuropathic pain, with keywords such as “valproic acid,” “topiramate,” “lamotrigine,” “epilepsy,” “seizure,” and “neuropathic pain.” (3) The blue cluster (169 keywords) reflects metabolically related pain manifestations, including abdominal pain and myalgia, resulting from metabolic disturbances such as ketoacidosis. Representative keywords included “diabetic ketoacidosis,” “metabolic acidosis,” “abdominal pain,” “myalgia,” and “thorax pain.” (4) The green cluster (165 keywords) focuses on central neuropathic pain and motor dysfunction associated with refractory epilepsy and status epilepticus, with keywords including “epilepsy,” “status epilepticus,” “neuropathy,” and “motor dysfunction.” All identified keywords are listed in Supplementary material S5. Together, these clusters demonstrate the diverse roles of the KD in pain management, serving both as a direct analgesic intervention and as a regulatory strategy in pain comorbid with neurological and metabolic disorders.

To further assess whether epilepsy-related research might obscure the identification of pain-related research hotspots, a supplementary sensitivity analysis was conducted by excluding epilepsy-related keywords (e.g., “epilepsy,” “seizure,” “convulsant”). The results showed that, after exclusion, the overall structure of the keyword co-occurrence network and the ranking of research hotspots remained largely unchanged. Themes such as migraine, chronic inflammatory pain, and neuropathic pain continued to form stable and well-defined clusters, indicating that epilepsy-related research did not mask the primary focus of ketogenic diet research in the context of pain. By integrating keyword clustering results from both databases, three main thematic directions were identified: (1) the direct analgesic effects of the KD on chronic inflammatory pain (e.g., osteoarthritis) and episodic disorders (e.g., migraine) through anti-inflammatory actions, metabolic regulation, and weight control; (2) the neuroprotective and therapeutic potential of the KD in neuropathic and central pain conditions, with mechanisms involving ketone body metabolism, modulation of oxidative stress, and synergistic effects with neuroactive drugs; and (3) the overall value and potential risks of the KD in improving pain-related emotional disorders, enhancing quality of life, and managing metabolic-related adverse effects such as ketoacidosis-related pain.

**Figure 7:**
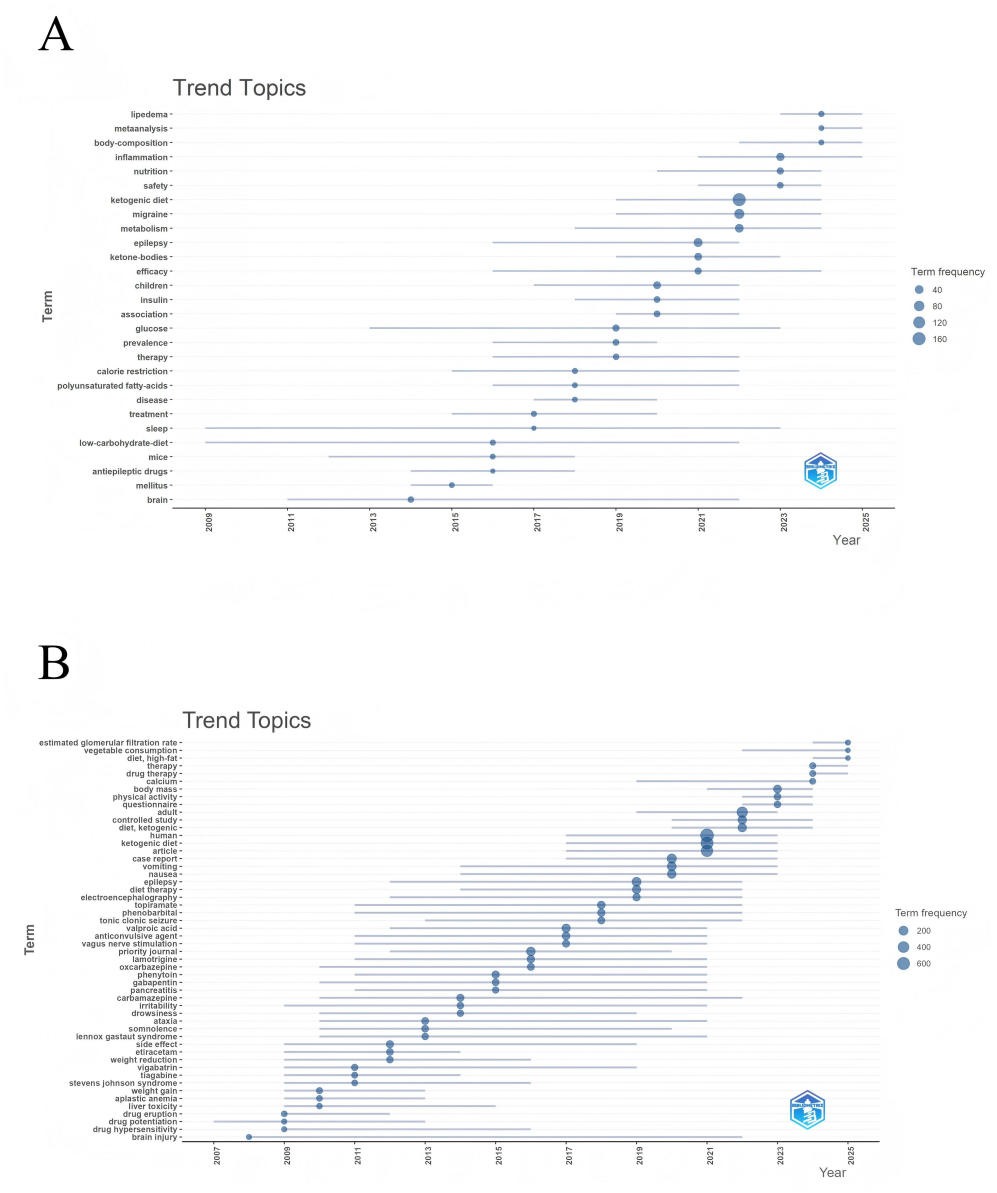
Trend topics on KD for pain. **(A)** Trend topics based on WoSCC. **(B)** Trend topics based on Scopus.

To predict future research trends, we constructed a dynamic thematic progression map using the bibliometrix package in R. WoSCC results (Figure 7A) showed a progressive evolution from metabolic foundations to clinical applications and, more recently, to precision-based development. Early studies (2006–2016) emphasized “low-carbohydrate diet,” “brain,” and “mice,” focusing on energy utilization and caloric restriction in experimental models. Mid-term studies (2016–2020) shifted to “therapy,” “ketone bodies,” “epilepsy,” and “treatment,” highlighting KD’s clinical efficacy in epilepsy, migraine, and related neurological disorders. Recent years (2020–present) have rapidly expanded to “ketogenic diet,” “migraine,” “pain,” and “metabolism,” further extending into “insulin resistance,” “lipedema,” “validation,” and “diagnosis,” reflecting a transition toward precision medicine in pain management, metabolic regulation, and diagnostic validation.

Scopus results (Figure 7B) demonstrated a thematic evolution from drug safety to dietary interventions and eventually to integrated management. Early studies (2006–2016) emphasized adverse effects of antiepileptic drugs, with keywords such as “drug eruption,” “weight gain,” “liver toxicity,” “vigabatrin,” and “side effect.” The mid-term period (2016–2020) focused on KD combined with pharmacological approaches, as reflected in terms like “diet therapy,” “electroencephalography,” “epilepsy,” “valproic acid,” and “vagus nerve stimulation.” More recently (2020–present), research has centered on “ketogenic diet,” “controlled study,” “follow-up,” “physical activity,” and “questionnaire,” underscoring KD-based interventions, long-term monitoring, and lifestyle management. Emerging terms such as “anxiety,” “therapy,” “drug therapy,” and “etiology” further suggest a shift toward multidimensional, comprehensive interventions and long-term outcome evaluations.

Collectively, findings from both databases reveal that research on KD and pain has progressed from early investigations into drug safety and metabolic regulation, to clinical applications in epilepsy and migraine, and is now advancing toward precision interventions and long-term follow-up strategies.

### 3.5 Integrated analysis of research hotspots

Synthesizing citation bursts, keyword clusters, and thematic evolution, three major hotspots were identified: (1) KD-based migraine strategies—modulating brain metabolism and excitability to reduce migraine frequency and intensity. (2) Broader chronic pain applications—extending efficacy beyond migraine to epilepsy, neuropathic pain, and other chronic pain conditions. (3) Ketone-mediated anti-inflammatory and neuroregulation—with BHB as a key modulator of inflammasome activity, neurotransmission, and mitochondrial function.

## 4 Discussion

### 4.1 General information

In this study, we constructed a comprehensive dataset comprising 328 articles from the WoSCC database and 786 articles from the Scopus database published between 2006 and 2025. The results showed a sustained increase in annual publications on KD–related pain research from 2006 to 2021, followed by a high-level but fluctuating pattern between 2022 and 2025. This trend indicates that the field experienced rapid growth in its early stage and has gradually entered a relatively stable phase in recent years. The United States has emerged as the leading contributor in this research area, with publication outputs markedly exceeding those of other countries. This dominance likely reflects the advantages of strong foundational neuroscience research, supportive research policies, well-established clinical translation systems, and a tradition of interdisciplinary collaboration. At the institutional level, the University of Basel (Switzerland) and Sapienza University of Rome (Italy) were the most productive institutions and served as key hubs within international collaboration networks. A total of 328 articles were published across 208 journals in WoSCC, while 786 articles were distributed among 426 journals in Scopus. Nutrients published the largest number of articles and received extensive citations, whereas Cephalalgia was the most highly cited journal. In addition, Nutrients and Epilepsia functioned as central nodes within the journal co-citation network. Collectively, these findings highlight the pivotal roles of Nutrients, Cephalalgia, and Epilepsia in the field of KD and pain research, serving as core journals and major platforms for the dissemination of related scientific advances.

### 4.2 Research hotspots and emerging trends

By integrating literature clustering, keyword frequency analysis, co-occurrence mapping, and thematic evolution, three major research hotspots were identified in the KD–pain field.

#### 4.2.1 KD as a preventive strategy for migraine

Migraine, a chronic neurovascular disorder, is characterized by recurrent moderate-to-severe headaches with high disability and reduced quality of life (20). As a non-pharmacological intervention, KD has gained growing evidence-based support for migraine prevention (21, 22).

Clinical studies consistently demonstrate KD’s effectiveness in drug-resistant migraine. Bongiovanni et al. showed that after 3 months of KD, median monthly headache days decreased from 30 to 7.5, with reductions in both headache duration and analgesic use (23). Lovati et al. reported that KD reduced headache frequency and intensity more effectively than a low-carbohydrate diet, with therapeutic benefits positively correlated with blood ketone levels (24). Valente et al. further found that KD decreased migraine days and medication use independently of weight loss (25). In a comparative study, Tereshko et al. evaluated three protocols (2:1 KD, low–glycemic index diet, very-low-calorie KD), all of which significantly reduced headache frequency, pain intensity, and disability (26).

Innovative dietary models, such as the Mediterranean–KD, have also been proposed. Olivito et al. demonstrated that combining classical KD with polyphenol- and omega-3–rich Mediterranean elements significantly reduced migraine attacks within 4–8 weeks and improved insulin sensitivity (27). Benefits have also been observed in younger populations: Pasca et al. reported that KD alleviated migraine in adolescents and improved sleep quality, suggesting broader quality-of-life improvements (28).

Mechanistically, KD’s efficacy is linked to BHB, which inhibits NLRP3 inflammasome activation—a key driver of neurogenic inflammation (12). By shifting cerebral energy metabolism toward ketone utilization, KD corrects energy deficits in the brain (7). Preclinical evidence also shows that KD reduces calcitonin gene-related peptide (CGRP) release from trigeminal neurons, thereby interrupting pain signaling (29). Additionally, ketones enhance GABAergic transmission while reducing glutamatergic excitability, elevating the threshold for cortical spreading depression (CSD), a major trigger of migraine (30).

In summary, KD improves cerebral energy homeostasis, reduces neuronal hyperexcitability, and raises CSD thresholds, thereby lowering migraine frequency and severity (31). Although large-scale randomized trials are needed to confirm long-term adherence and safety, current evidence supports KD as a viable component of comprehensive migraine management.

#### 4.2.2 Broader applications of KD in chronic pain disorders

The KD has gained increasing attention in recent years for its role in chronic pain management. Beyond the emerging evidence supporting its preventive efficacy in migraine, KD has also demonstrated considerable therapeutic potential in epilepsy, neuropathic pain, and other chronic pain conditions (20, 32, 33). This cross-disease exploration highlights KD as a promising non-pharmacological option for the comprehensive management of chronic pain (14, 31, 33).

Beyond migraine, KD has shown therapeutic promise across multiple chronic pain conditions. In epilepsy—the condition with the strongest evidence base—long-term KD use not only reduces seizure frequency but also improves comorbid pain and quality of life (34). These findings provide a foundation for extending KD to other pain disorders.

In neuropathic pain, preclinical studies show that KD alleviates hyperalgesia by modulating both peripheral and central pain mechanisms (35–37). Randomized controlled trials further suggest that KD reduces neuroinflammation and central sensitization, leading to pain relief (38).

KD has also been investigated in inflammatory pain conditions. Clinical reports suggest benefits for patients with rheumatoid arthritis, fibromyalgia, and multiple sclerosis, including pain reduction, fatigue improvement, and enhanced quality of life (39–41).

Overall, KD demonstrates cross-disease analgesic potential spanning epilepsy, neuropathic pain, and inflammation-related conditions, offering a promising non-pharmacological alternative for patients with refractory pain. However, current evidence remains limited, as most studies are small-scale or preclinical. Large, multicenter randomized controlled trials are needed to validate efficacy, assess long-term safety, and establish individualized treatment protocols for chronic pain management.

#### 4.2.3 Ketone-mediated anti-inflammatory and neurofunctional mechanisms

Ketone bodies, particularly BHB, are increasingly recognized as signaling molecules with diverse regulatory effects beyond their role as alternative energy substrates.

First, BHB exerts potent anti-inflammatory effects. As an endogenous inhibitor of class I and II histone deacetylases (HDACs), it upregulates antioxidant and stress-resistance genes such as FOXO3a and SOD2, thereby mitigating oxidative stress—a shared driver of chronic pain and neurodegeneration (42). BHB also directly inhibits NLRP3 inflammasome activation, preventing IL-1β and IL-18 maturation and release (12). Additionally, it activates GPR109A (HCA2), which suppresses macrophage-mediated inflammatory responses (43).

Second, BHB modulates neurotransmission by enhancing GABA synthesis and signaling while suppressing glutamate release, thus restoring excitatory–inhibitory balance (44). This mechanism reduces central sensitization and neuronal hyperexcitability (45). During ketosis, glutamate can also be partially converted to GABA, further reinforcing inhibitory tone (6).

Third, BHB enhances mitochondrial function and bioenergetics. It improves respiratory chain efficiency, reduces reactive oxygen species, and stabilizes ATP production, thereby supporting neuronal survival (46). BHB also activates PGC-1α, promoting mitochondrial biogenesis and antioxidant defenses (47, 48).

Finally, KD has been shown to increase adenosine levels, which act on A1 receptors to provide neuroprotective and analgesic effects (14).

Collectively, ketone bodies orchestrate a multifaceted regulatory network involving anti-inflammatory signaling, neurotransmitter balance, and mitochondrial bioenergetics, ultimately mediating KD’s analgesic and neuroprotective effects. These mechanisms provide a strong rationale for clinical applications of KD and exogenous ketone supplementation in pain management.

### 4.3 Differences between the WoSCC and Scopus databases and their impact on the identification of research hotspots

In this study, both the WoSCC and Scopus databases were included for bibliometric analysis to systematically characterize the overall knowledge structure and developmental trajectory of research on the KD and pain from complementary perspectives. Notably, this study did not assume equivalence between the two databases in terms of literature volume, disciplinary coverage, or research types. Instead, acknowledging their long-standing structural differences, a dual-database strategy was deliberately adopted to enhance the comprehensiveness and robustness of the findings.

In the present analysis, the number of relevant publications indexed in Scopus was substantially higher than that in WoSCC (786 vs. 328). This discrepancy can largely be attributed to systematic differences in journal selection criteria and disciplinary coverage between the two databases. WoSCC places greater emphasis on journal academic impact and stability, preferentially indexing studies published in high-impact journals with strong theoretical foundations and methodological rigor. In contrast, Scopus provides broader coverage of clinical medicine, public health, and applied research, and shows greater inclusiveness toward publication types such as clinical studies, observational research, and case reports. Given the inherently clinical orientation of pain research and the fact that KD interventions are often first explored through small-scale clinical studies or case reports, the larger volume of literature captured by Scopus is therefore expected. These database-specific characteristics were also reflected in the keyword analysis. Compared with WoSCC, the Scopus dataset contained a higher frequency of keywords related to clinical practice, case observations, and study types (e.g., clinical article, case report), and exhibited more fine-grained terminology related to migraine, metabolism-related pain, and dietary intervention strategies (Table 7; Figure 6B). This pattern does not indicate a shift in research themes, but rather highlights the advantage of Scopus in capturing real-world clinical research and early exploratory studies.

Despite differences in publication volume and keyword composition, a high degree of consistency was observed between the two databases with respect to overarching research directions and core themes. Integrated analyses revealed that the application of the KD in specific pain syndromes such as migraine, its potential anti-inflammatory and metabolic regulatory mechanisms, and its clinical value as a non-pharmacological strategy for pain management were repeatedly identified in both databases. The primary differences lay in research emphasis: WoSCC data more prominently reflected theoretical foundations and mechanistic investigations, whereas Scopus data expanded coverage of clinical applications and practice-oriented research.

Accordingly, rather than treating results from the two databases as interchangeable, this study conducted parallel analyses to enable cross-validation and complementary identification of research hotspots. This dual-database approach helps mitigate structural bias associated with reliance on a single data source, allowing the study to capture both the core scientific questions and the evolving clinical applications of KD research in pain. As a result, the reliability and interpretability of the study’s conclusions are strengthened.

### 4.4 Implications for pain research paradigms and the development of non-pharmacological intervention frameworks

Integrated bibliometric analysis of the WoSCC and Scopus databases indicates that research on KD–based pain interventions has evolved into a relatively stable and expanding research domain. This trend reflects not only growing interest in a specific dietary intervention, but also a broader shift in pain research paradigms toward non-pharmacological and systems-oriented approaches.

From the perspective of knowledge structure evolution, the sustained growth of KD–related studies suggests a gradual transition in pain research from traditional frameworks focused on isolated neural or inflammatory mechanisms toward an integrative “metabolic–functional” perspective. Metabolic regulation and systemic homeostasis are increasingly recognized as relevant dimensions in understanding pain persistence and modulation.

Analysis of research types reveals that studies on KD and pain are gradually shifting from exploratory observations toward more hypothesis-driven investigations, although the overall evidence base remains underdeveloped. Substantial heterogeneity in study design and outcome measures persists, underscoring a common methodological challenge in non-pharmacological analgesic research.

Importantly, recent research trends suggest an increasing emphasis on integrating KD interventions into broader multimodal pain management frameworks rather than evaluating them as isolated treatments. As a lifestyle-based intervention with systemic regulatory properties, its potential value lies in multi-pathway modulation of pain-related functional states, particularly in chronic pain contexts.

To strengthen the evidence base, future studies should adopt more objective and standardized pain assessment paradigms. Integrating subjective pain scales with quantitative sensory testing, neurophysiological or imaging markers, and functional or metabolic indicators may improve the objectivity and comparability of non-pharmacological pain research.

At the policy and research planning level, the growing body of evidence on KD–based pain interventions aligns with global strategies emphasizing non-pharmacological pain management and reduced long-term medication dependence. These findings support the inclusion of dietary and lifestyle interventions in future pain research agendas and clinical management frameworks.

### 4.5 Limitations

This study systematically examined KD and pain research using WoSCC and Scopus, offering a comprehensive overview of publication trends, hotspots, and thematic evolution. Nonetheless, several limitations should be noted. First, restricting the analysis to WoSCC and Scopus may have excluded studies indexed elsewhere, thereby limiting comprehensiveness. Second, only English-language publications were included, excluding potentially relevant studies published in other languages. While English dominates academic publishing, this unavoidably narrows the global perspective. Third, due to restrictions in Scopus export formats, co-citation analysis was performed only with WoSCC data, limiting the breadth of knowledge-base mapping. Despite these limitations, the findings remain robust and provide valuable insights for guiding future research on KD and pain.

## 5 Conclusion

This study provides an in-depth analysis of the major research hotspots and emerging trends in applying the KD for pain management. The key findings are as follows:

- The field has experienced sustained growth followed by a period of high-level fluctuation. The United States has emerged as the dominant research hub. The University of Basel (Switzerland) and Sapienza University of Rome (Italy) rank among the leading publishing institutions and serve as central nodes in the international collaboration network.
- Nutrients has published the largest number of articles and received extensive citations. Cephalalgia is the most frequently cited journal, while Nutrients and Epilepsia serve as central hubs for collaboration. Collectively, these findings highlight the pivotal roles of Nutrients, Cephalalgia, and Epilepsia as key platforms for disseminating KD-related pain research.
- Research hotspots are concentrated in three areas: (1) ketogenic dietary interventions for migraine; (2) the application of KD in diverse chronic pain conditions; and (3) ketone body–mediated mechanisms of anti-inflammation and neuroregulation.
- Research trends have shifted from early investigations on drug safety and metabolic effects to clinical applications in epilepsy and migraine, and are now advancing toward multidimensional precision interventions and long-term follow-up studies.

In summary, this study systematically mapped the research trends and hotspots in KD-based pain interventions, offering valuable insights for the field. These findings not only provide researchers with essential background knowledge on current progress but also lay a foundation for exploring novel directions. By identifying existing frontiers and potential priority areas, this study offers strong guidance and support for future innovative research.

## Data Availability

The datasets presented in this study can be found in online repositories. The names of the repository/repositories and accession number(s) can be found in the article/Supplementary material.

## Footnotes

1
http://www.bibliometrix.org
